# Nutritional Behaviors, Health Literacy, and Health Locus of Control of Secondary Schoolers in Southern Poland: A Cross-Sectional Study

**DOI:** 10.3390/nu13124323

**Published:** 2021-11-29

**Authors:** Mariusz Duplaga, Marcin Grysztar

**Affiliations:** Department of Health Promotion and e-Health, Institute of Public Health, Faculty of Health Sciences, Jagiellonian University Medical College, 31-066 Krakow, Poland; marcin.grysztar@uj.edu.pl

**Keywords:** nutritional behaviors, health literacy, multidimensional health locus of control, secondary school students

## Abstract

Nutritional behaviors remain an essential part of a healthy lifestyle. It seems obvious that unfavorable health behaviors adopted in adolescence are maintained late in adulthood and may have a profound effect on health status. The main aim of this study was to assess the association between nutritional behaviors and health literacy (HL), health locus of control (HLC), and socioeconomic variables in secondary school pupils from a voivodship (the main unit of territorial division) in southern Poland. The analysis was based on dataTable from a paper-and-pencil survey taken by 2223 pupils from schools selected as the result of cluster sampling. The survey questionnaire encompassed a set of five items asking about dietary patterns and the consumption of fruit and vegetables as well as fast food, a European Health Literacy Project Questionnaire consisting of 47 items, the Multidimensional Health Locus of Control (MHLC) scale, and items asking about sociodemographic and economic variables. Uni- and multivariate logistic regression models have been developed to assess the predictors of indicator nutrition behaviors. The adjusted models revealed that internal HLC was not significantly associated with any of analyzed nutritional behaviors. “Powerful other HLC” and “Chance HLC” (dimension of external HLC) were significant predictors of the selected dietary patterns. Furthermore, higher HL was associated with higher consumption of fruit and vegetables [odds ratio, 95% confidence interval (OR, 95% CI)]: 1.02 (1.01–1.04) and with lower consumption of fast food (OR, 95% CI, 0.98, 0.95–0.999). There was a significant relationship between gender, the size of the household, self-assessed economic situation, expenditures on mobile phones, and weekly duration of Internet use and selected nutrition behaviors. In conclusion, developed regression models confirmed a significant relationship between HL and the types of consumed food, but not with dieting patterns. Contrary to earlier studies, internal HLC was not associated with nutrition behaviors. In our study, boys showed more favorable nutritional behaviors than girls. More intense use of the Internet was associated with less beneficial nutritional behaviors. This study brings important results that should have an impact on health promotion interventions addressed to adolescents in southern Poland.

## 1. Introduction

Adolescence is a transitory period between childhood and adulthood. As stated by the Lancet Commission on Adolescent Health and Wellbeing, this period is recognized as the healthiest time of life [1]. However, health problems and risk factors occurring in adolescence have a profound effect on life-time health [1]. It is also clear that unfavorable health behaviors initiated during adolescence persist in adulthood. As adolescence is characterized by rapid growth, adequate nutrition is of upmost importance for achieving full growth potential [2]. In adolescence, health behaviors may be influenced by many factors. This particularly applies to nutrition. Among factors with a considerable impact on the nutritional behaviors of adolescents, Das et al. list peers’ opinions, parental example, food availability, food preferences, cost, convenience, personal and cultural beliefs, mass media, and finally, the perception of body image [2]. Inadequate dietary habits may lead to many chronic diseases including obesity and its consequences. Moreno et al. underline the importance of dietary factors in the development of obesity, including meal frequency and distribution, snacking, skipping meals, sweetened beverage consumption, size of the food portions, eating away from home, and the consumption of fast food [3].

According to the theory proposed by Cockerham, health lifestyles are “collective patterns of health-related behaviors based on choices from options available to people according to their life chances” [4]. Life chances, described by Cockerham as “structure”, encompass such factors as class circumstances, age, gender, race/ethnicity, collectivities (defined as collections of actors linked together through particular social relationships), and living conditions. In turn, life choices or agency mean the ability to manage one’s life and are related to socialization and experience. Furthermore, the interplay of choices and chances leads to dispositions toward given behaviors or actions, which Cockerham names “habitus”. According to this theory, health behaviors in adolescence and adulthood are not distributed randomly in society. Today, it seems obvious that socioeconomic status is one of the most important determinants of health behaviors in adulthood. Sex and ethnicity are among other key factors in the formation and maintenance of health lifestyles in adulthood [4]. Burdette et al. reported, having analyzed the data of the National Longitudinal Study of Adolescence to Adult Health, that socially patterned lifestyles can already be observed in adolescence and remain related to the distribution of physical health across the early life course [5].

Many definitions of HL have been formulated. In this paper, we cite the one developed by Sörensen et al. within the European Health Literacy Survey (EHLS) Project. It was at the core of the multidimensional concept of HL further used to develop an instrument used in our study. This definition says that HL should be understood as “the knowledge, motivation and competences to access, understand, appraise and apply health information in order to make judgments and take decisions in everyday life concerning health care, disease prevention and health promotion to maintain or improve quality of life throughout the course of life” [6]. The researchers working in the EHLS Project developed a questionnaire containing (in its basic version) 47 items (HLS-EU-Q47) assessing self-reported competencies in performing actions related to handling health information in three domains: healthcare, disease prevention, and health promotion [7]. A survey performed within the EHLS project in eight European countries showed that in the population 15 and older, the correlation between HL and health behaviors, apart from smoking habits, is significant but rather weak [8].

The relationship between HL and health behaviors in adolescents has been analyzed by several authors. Fleary et al. in a systematic review published in 2018 identified 17 studies focused on such analysis [9]. In 13 studies, a significant relationship between HL and “health-promoting behaviors” regarding alcohol use, tobacco use, medical adherence, health-related information seeking, and risky sexual behaviors has been confirmed. According to Levin-Zamir et al., media HL was positively associated with a high score in terms of health-promoting behavior, including nutritional and dietary habits [10]. In turn, Chang et al. observed that functional HL was a predictor of the total score regarding health-promoting behavior, based on combinations of behaviors including nutrition [11]. Buja et al. conducted a systematic review to assess the association between HL and dietary intake of sugar, fat, and salt [12]. They reported that the identified studies have not confirmed a significant association between HL and salt (2 cross-sectional studies) or fat intake (one study only). In turn, the relationship between a higher HL and lower sugar intake was reported by three out of the seven eligible studies; in one study this association occurred only in males, and in two studies there was no association.

Klinker et al. observed that in students from vocational education and training schools in Denmark, lower HL, as measured by the Health Literacy Questionnaire (HLQ), is associated with unhealthy behaviors, including a lower frequency of having breakfast on weekdays (used as an indicator of nutritional behavior) [13]. Recently, Bektas et al. reported that HL and self-efficacy were significant predictors of health behaviors, including nutrition, in adolescents from 13 to 18 years old in Turkey [14]. According to Chrissini & Panagiotakos [15], HL may be treated as a determinant of childhood and adult obesity. Interestingly, Nagy-Pénzes et al. found no association between health-related knowledge and favorable health behaviors. The authors suggested that adolescents are more influenced by their living context and that sufficient health-related knowledge does not suffice to shape health behaviors. Translating these findings into the concepts proposed by Cockerham, it seems that structure shaping factors prevail over agency in this age group [16].

The construct of the “health locus of control” shows “whether individuals believe their health status is under their own control … or is under the control of forces external to themselves, such as other people, fate, luck, chance, or ‘a higher power’” [17]. In the 1960s, Rotter proposed the social learning theory, stating that people may present with an internal or external locus of control (I/E dimension) [18]. Following this theory, Walston et al. developed (initially in 1976) the Health Locus of Control (HLC) scale, assuming that locus of health control is a unidimensional concept [19]. However, the first studies with the HLC scale tended to show that internality and externality do not reflect one dimension. The next version of the scale, the Multidimensional Health Locus of Control (MHLC) scale [20], was prepared in reference to the work of Levenson, who questioned conceptualizing the locus of control as a unidimensional construct [21]. Instead of one dimension, Levenson divided the I/E dimension into three separate dimensions: internal and two external, “Powerful Other” and “Chance” [21]. Persons with higher internal locus of control are more likely to assume that their health depends on their behavior and remains within their control. External health locus of control is associated with the conviction that external factors are perceived as responsible for control; in the case of “powerful other health locus of control”, a person believes that other people are in control of their health, and in the case of “chance locus of control”, it is associated with chance factors. The MHLC scale includes three subscales allowing assessment of the degree that people believe their health to depend on their own actions (internal HLC, IHLC, subscale), the actions of a “powerful other” (PHLC subscale) or to result from chance (CHLC subscale) [17]. The MHLC scale has been used in many studies assessing the determinants of health behaviors. Consistently, observations indicate that high scores achieved on the IHLC subscale are associated with higher medication adherence and that high results in PHLC and CHLC are associated with medication non-adherence [22]. However, the results of the analysis of the relationship between MHLC scores and healthy lifestyle behaviors are variable. A significant association was reported for MHLC and dietary patterns [23], body mass index (BMI) [24], quality of life in patients with diabetes [25], the effect of organic food label on food consumption [26], compliance with childhood vaccinations [27], self-assessed health [28], cyberbullying and being victimized [29], and even for mortality [30]. The studies performed in Polish populations revealed a significant association between IHLC and health behaviors, including health nutrition, physical activity and using safety belts in cars, in students of health sciences [31], between PHLC and health behaviors in medical workers [32], and between IHLC, PHLC, and health behaviors in adults from other professional groups [32]. Mazur et al. reported a significant correlation between HL as measured with Health Literacy for School-Aged Children (HLSAC) and IHLC and PHLC subscale scores in a group of junior high school students.

It should be noted that the relationship between HL and health behaviors, and specifically dietary patterns, in Polish adolescents have not been studied before. Therefore, based on a large sample of secondary schoolers from southern Poland, we aimed to analyze the relationship between eating behaviors and HL and MHCL.

## 2. Materials and Methods

### 2.1. Survey

The analysis was carried out on data from a “paper-and-pencil” survey completed by a sample of pupils of secondary schools located in the Małopolska Voivodship (the main unit of territorial division). Cluster two-stage random sampling was performed. First, a list of 20 secondary schools was generated from the inventory of the Board of Education. The directors of the selected schools were approached and asked for the permission to perform the survey. The invitations were accepted by 9 schools. In each school, depending on its size, not less than five and not more than ten classes of various grades and profiles were randomly selected. Before initiating the survey in a school, the parents of the pupils from the selected classes were informed about the aims and the methodology of the study. Then, pupils from these classes were informed about the survey and asked for their informed consent. Informed consent was also requested from the parents or legal guardians of pupils younger than 18 years old. The respondents were also advised that at any moment they could withdraw from the survey. The surveys were conducted in the selected schools from September to October 2017.

The questionnaire used in the survey consisted of 130 items. Apart from the Polish version of the 47-item HLS-EU-Q47 [6] and the 18-item MHLC scale [20,33], it included a set of questions asking about respondent health behaviors and items exploring their socioeconomic status. The Polish version of the HLS-EU-Q47 was obtained from the EHLS Project team. The version of the MHLC scale used in the survey was translated to Polish by Juczyński [34].

The Bioethical Committee of Jagiellonian University issued an agreement for the study on 25 September 2014 (decision No KBET/193/B/2014).

### 2.2. Dependent Variables

Dichotomized variables, reflecting nutritional routines and practices, were used as dependent variables in uni- and multivariate logistic regression models. They were established based on responses to the items asking about the daily number of meals (at least four or fewer than four), the largest meal (dinner or other), meal regularity (regular or not regular), the consumption of fruit and vegetables (at least once daily or less often than once daily) and the consumption of fast food (at least a few times per month, less frequently, or not at all). The questionnaire used in the survey was developed for the assessment of an array of health behaviors. Therefore, we have not been able to perform a detailed assessment of the dietary patterns and nutritional choices of the respondents. The selection of five items was a matter of the compromise, attempting to provide indicator variables for respondent nutritional behaviors. Furthermore, the specific items have been selected after taking into consideration national guidelines [35,36] and previous studies performed in adolescents [37,38,39,40]. The final decision was arbitrary but to some extent informed by the frequency of use of specific items in other studies in Poland. The dichotomization of the variables was based on the guidelines [36] that at least four meals are recommended per day, that dinner should provide 30–40% of the daily caloric supply [35], and that meals should be as regular as possible [35,36]. The consumption of fruit and vegetables and fast food have been treated as an indicator of nutritional behaviors in relation to the selection of healthy and unhealthy food.

### 2.3. Independent Variables

The variables assessed as potential predictors in regression models reflected

- sociodemographic characteristics: sex, attended grade in school (as an equivalent of age), parental levels of education, marital status of parents, place of residence, number of household members, type of school (providing general or vocational education).
- family and respondent economic status: monthly mobile phone expenses, self-assessed family economic situation, and obtaining external, financial or material help.
- weekly duration of Internet use.
- general HL score.
- MHLC subscale scores (IHLC, PHLC and CHLC).

The HL score was calculated in line with the recommendations of the EHLS Project [41]. Responses to each item of the HLS-EU-Q47 were converted to numerical values from 1 to 4. If the respondent was not able to provide an opinion, such a response was treated as missing values. The general score was calculated only for those respondents who responded to at least 80% of the items in the questionnaire. The formula used for the development of the general HL score was as follows: (mean-1) × (50/3). In the result, the initial mean of individual item scores was transformed to a value ranging from “0” (for the lowest possible score) to “50” (for the highest possible score).

MHLC subscale scores were calculated according to the guidelines of Wallston et al. [20]. Participants responded to items included in the MHLC scale using a 6-point Likert scale, from strongly disagree to strongly agree. These responses were transformed into numerical values from 1 to 6. Each subscale consists of six items. Subscale scores were calculated as sums of individual scores and could range from 6 to 36. As types of control are not mutually exclusive, all three scores were applied in multivariate logistic regression models.

### 2.4. Statistical Analysis

Statistical analysis was performed with IBM SPSS Statistics v.26 software (IBM Corp. Armonk, NY, USA). The mean and standard deviation (SD) were provided for continuous variables and relative and absolute frequencies for categorical variables.

For the variables used as indicators of dietary routines and habits, both uni- and multivariate logistic regression models were developed. The Hosmer and Lemeshow chi^2^ test and the Nagelkerke R square were calculated for each model. The odds ratio (OR), 95% confidence interval (95% CI) and p-values were presented for univariate models, and adjusted values of OR (aOR), 95% CI (a95% CI) and *p*-value presented for independent variables. Statistical significance was assumed at the level of *p* < 0.05.

## 3. Results

### 3.1. Characteristics of the Study Group

The percentage of potential participants who refused to participate in the survey was 4.56% (n = 107). The final number of questionnaires included in the analysis was 2223. The mean age of respondents was 17.01 (SD = 0.97). In the study group, 33.71% were male (n = 731). A total of 17.69% of the respondents were from secondary schools providing vocational education (n = 393). Table 1 shows detailed characteristics of the study group.

### 3.2. Daily Number of Meals

In the study group, 70.16% of the respondents consumed at least four meals daily as, recommended. Univariate logistic regression analysis revealed that the daily number of meals is significantly associated with PHLC and CHLC subscores on the MHLC scale, the type of school, education level of mother, and place of residence (please see Table S1). Respondents with higher PHLC and CHLC scores are less likely to consume at least four meals per day (OR, 95% CI: 0.89, 0.81–0.99, and 0.83, 0.75–0.92, respectively). Students attending vocational schools have a 23% lower likelihood of consuming an adequate number of meals daily. Furthermore, pupils living in urban areas with a population above 200,000 are about 40% less likely to consume the recommended number of meals than those living in rural areas (OR, 95% CI: 0.62, 0.49–0.77). Finally, the respondents whose mothers completed secondary education are significantly more likely to have at least four meals per day than those whose mothers did not complete secondary education (OR, 95% CI: 1.27, 1.001–1.61).

A multivariate model of logistic regression performed with all independent variables analyzed in univariate models showed some additional interesting findings (Table 2). The inclusion of PHLC and CHLC subscores in one model resulted in a reversal of the relationship between PHLC and dependent variable. The respondents with higher PHLC were more likely to consume at least four meals per day than those with lower PHLC (OR, 95% CI: 1.17, 1.02–1.34). Furthermore, it turned out that males are more likely to consume daily more meals than females (OR, 95% CI: 1.34, 1.06–1.68). Finally, the type of the school lost its effect on the dependent variable (OR, 95CI: 0.77, 0.58–1.02).

### 3.3. Regularity of Meals

Only 30.1% of respondents stated that they consumed meals regularly. In univariate models, a significant relationship with the dependent variable reflecting having regular meals is observed for all subscores of health locus of control, gender, type of school, level of father’s education reached, place of residence, monthly expenditures on mobile phones and weekly amount of Internet use (please see Table S2). A higher internal health locus of control (IHLC) was associated with higher odds (OR, 95% CI: 1.17, 1.04–1.33) and higher subscores reflecting the influence of other people (PHLC) and chance (CHLC) with lower odds of having regular meals (OR, 95% CI: 0.89, 0.81–0.99, and 0.75, 0.67–0.83, respectively). More regular meals were also observed in boys than girls (OR, 95% CI: 1.41, 1.16–1.71). Less regular meals were reported by students from schools providing vocational training than from schools with general education (OR, 95% CI: 0.64, 0.49–0.83), the residents of urban areas with population >200,000 than residents of rural areas (OR, 95% CI: 0.76, 0.60–0.96), the respondents spending the most on their mobile phones than those spending the least (OR, 95% CI: 0.58, 0.37–0.92) and those using the Internet most intensively than those using it for the shortest amount of time in the week (OR, 95% CI for comparison between Internet use for >35 and >21–35 h: 0.62, 0.43–0.91, and 0.45, 0.31–0.66, respectively). Interestingly, not the mother’s, but the father’s level of education was significantly associated with having regular meals (OR, 95% CI for comparison between fathers with university and secondary education and those with lower than secondary education: 1.27, 1.02–1.58, and 1.35, 1.07–1.70, respectively).

The multivariate regression model confirmed the relationships found in univariate models for gender, type of school, place of residence, monthly expenditures on mobile phone and weekly amount of Internet use (Table 2). Analogically as in the analysis for the dependent variable reflecting the number of meals per day, the reversal of the effect of PHLC was observed (OR, 95% CI: 1.16, 1.01–1.33). In the multivariate model, the number of household members became a significant predictor. The respondents living in household with 4 and 5 members were less likely to have regular meals than those from households with not more than 3 household members (OR, 95% CI: 0.68, 0.50–0.92, and 0.67, 0.47–0.94, respectively).

### 3.4. The Largest Meal

For 84.84%, dinner was the largest meal which is in line with the recommendation that it should provide the largest portion of the caloric supply during the day [35,36]. Univariate modelling showed that the dependent variable indicating the largest daily meal was significantly associated with PHLC, gender, the grade at school and the number of household members (Table S3). The respondents with lower PHLC (OR, 95% CI: 0.89, 0.81–0.99) and from the oldest grades, as opposed to the lowest grade, were less likely to have dinner be the largest meal (OR, 95% CI: 0.74, 0.56–0.98). For males more frequently than females (OR, 95% CI: 1.30, 1.001–1.68) and the pupils from households with 4 or more than 5 members (OR, 95% CI: 1.54, 1.12–2.12, and 1.54, 1.09–2.18, respectively) dinner was the largest meal. The multivariate regression model confirmed significant associations for gender, the grade attended at school, and the number of household members (Table 3). Additionally, it appears that a significant relationship was revealed for the type of school (OR, 95% CI: 0.59, 0.42–0.84) and self-assessed economic status (OR, 95% CI for the comparison between the respondents reporting their economic situation as good and as worse than good: 1.49, 1.01–2.18).

### 3.5. The Consumption of Fruit and Vegetables

The percentage of the respondents who consumed fruit and vegetables less frequently than once daily was very high (42.19%). Univariate analysis showed that fruit and vegetables were consumed at least once daily more often by the respondents with higher rather than lower HL (OR, 95% CI: 1.03, 1.01–1.04), by those whose mother or father attained at least secondary education, and the students self-assessing their economic situation as very good rather than worse than good (OR, 95% CI: 1.58, 1.21–2.08) (please see Table S4). Pupils from schools providing vocational training (OR, 95% CI: 0.59, 0.47–0.73) and those using the Internet more than 35 h per week (OR, 95% CI: 0.60, 0.43–0.84) consumed fruit and vegetables less frequently. Additionally, the respondents with higher PHLC and CHLC subscores were less likely to eat fruit and vegetables at least once daily.

The multivariate model confirmed a significant relationship for HL, the type of school, the level of father’s education and the amount of time spent on the Internet per week (Table 4). Interestingly, the relationship between mother’s level of education was no longer significantly associated with eating fruit and vegetables. The relationships between dependent variables and health locus of control were not significant either, but a significant relationship emerged for monthly expenditures on mobile phone (OR, 95% CI for the comparison between those spending the most and the least: 0.6, 0.36–0.98).

### 3.6. The Consumption of Fast Food

Nearly 86% of study participants consumed fast food at least a few times monthly. Univariate regression revealed a significant association between PHLC and CHLC subscores (OR, 95% CI: 1.56, 1.35–1.81 and 1.36, 1.19–1.57, respectively), the grade at the school (OR, 95% CI for comparison between the respondents from the highest grades and lowest grade 0.69, 0.52–0.92), and receiving external support (OR, 95% CI: 0.74, 0.58–0.96) (please see Table S5). Furthermore, the pupils who declared the highest expenditures on mobile phone were more than two times more likely than those spending the least to eat fast food at least several time per month (OR, 95% CI: 2.21, 1.28–3.81). Consistently, all pupils using the Internet longer than 2 h per week were 2–4 times more likely to more frequently consume fast food. In the multivariate model, the significant association between the CHLC subscore and fast-food consumption disappeared, but HL became a significant predictor (OR, 95% CI: 0.98, 0.95–0.999) (Table 4). Higher PHLC remained a predictor of a higher likelihood of fast-food consumption. Furthermore, variables related to expenditure on mobile phones and weekly use of the Internet, as well as receiving external support, maintained their relationships with fast-food consumption.

## 4. Discussion

In our study, we have analyzed the determinants of nutritional behaviors among high school students from the Małopolska Voivodship in Poland. The indicator behaviors included in the analysis were the number of meals consumed daily, the regularity of meals, the largest meal each day, as well as the frequency of the consumption of fruit and vegetables and fast food. Apart from socioeconomic variables, HL and HLC have been used in logistic regression models as independent variables.

We have observed that in multivariate models, HLC was a significant predictor of three, and the level of HL, of only two out of the five indicator behaviors. Interestingly, the HL score measured with the HLS-EU-Q47 instrument was only significantly associated with the consumption of fruit and vegetables and fast food. The respondents with higher HL showed a higher frequency of consuming fruit and vegetables and lower frequency of consuming fast food.

The relationship between HL and health behaviors in children, adolescents and young adults has been analyzed in many studies. Chang et al. (2011) reported a significant relationship between HL measured with S-TOHFLA instrument and the Health Promotion Scale (HPS) in high school students in Taiwan [11]. The authors underlined that this relationship was particularly visible for the HPS subscale for nutritional behaviors. The participants with low HL showed about a 40% lower likelihood of beneficial behavior than those with high HL. Levin-Zamir et al. observed that medial HL is significantly associated with the health behavior score. In this study, nutritional behaviors were assessed with three variables: refraining from dieting practice, snacking and daily use of presweetened drinks. Klinker et al. (2020) analyzed the relationship between HL and health behaviors in students from vocational education and training schools in Denmark [13]. The consumption of breakfast on weekdays was used as an indicator of dietary behavior. The adjusted analysis showed that lower HL scores in two scales of the Health Literacy Questionnaire were associated with less frequent breakfasts in the study group.

A systematic review published in 2018 by Fleary et al. showed a meaningful relationship between HL and adolescent health behaviors; however, none of the identified studies analyzed nutritional behaviors individually [9]. In a few studies, a nutrition subscale was part of a combined instrument measuring health or health promotion behaviors.

A recent study by Ayaz-Alkaya & Kulakci-Altintas (2021) confirmed a positive correlation between HL and health nutrition-exercise behavior and meal pattern in students of grades 6–8 in Turkish school [42]. Another study performed among adolescents from Turkey confirmed the association between HL and subdimensions of health lifestyle behaviors, including nutrition [14]. The self-efficacy and HL levels explained as much as 20.1% of the nutrition sub-dimension score in regression analysis. A significant correlation was also reported between e-health literacy (eHL) and the nutrition subscale of the Adolescent Health Promotion Scale by Gurkan & Ayar [43].

The rather complex pattern of interrelations between the level of HL and nutritional behaviors requires further attention. The presence of a significant relationship between HL and variables reflecting nutritional choices in relation to fruit and vegetables and fast food, while no relationship with dietary patterns such as the regularity or number of meals, may expose gaps in health education activities addressed to children and adolescents at school.

In our study, only two dimensions of MHLC, PHLC, and CHLC were significantly associated with selected variables reflecting nutrition behaviors. The respondents with higher PHLC scores were more likely to have a recommended number of meals per day and keep the regularity of meals, but were also more likely to consume fast food. Higher CPHL scores were observed for the respondents who less frequently consumed a recommended number of meals per day, and showed lower regularity of meals. There was no significant association between IHLC score and nutrition behaviors.

Earlier studies have not yielded unequivocal results about the role of MHLC subdimensions and health behaviors. The review from 1997 published by Abu Sabha & Achterberg showed a lack of consistent relationship between the measures of locus of control or health locus of control and nutrition behavior in various groups of participants [44]. For example, the study of Raab et al. showed that frequent users of supplements had significantly higher IHLC and PHLC than those who used them rarely [45]. However, another study did not find such differences [46] (Read et al. 1991). The number of studies analyzing the relationship between MHLC and nutrition behaviors carried out in younger populations is not high. In the study from 2006, O’Dea & Wilson did not observe a significant relationship between dietary locus of control and BMI in children and adolescents [47]. In turn, the study by Gayathri et al. from 2011, revealed that more favorable behaviors reflected by a combined score (Global School Bases Health Assessment, GSBHA) were observed in adolescents from India with higher IHLC and PHLC subscores of MHLC values. However, the nutrition dimension of GSBHA did not significantly correlate with any MHLC subscale [48].

The study performed among University students from Germany showed that higher IHLC was associated with higher attention to healthy nutrition [49]. Marr & Wilcox reported that self-efficacy and social support mediated the relationship between IHLC and fruit and vegetable consumption among college students [50]. They also found that there was no significant relationship between IHLC and dietary fat intake. Hosseini et al. found that IHLC and PHLC scores were significantly associated with health-promoting behaviors measured on the Adolescent Health-Promoting Scale [51]. High CHLC showed an adverse effect on health behaviors. According to the authors, adolescents with higher IHLC had more favorable eating habits. Interesting results on parent and child locus of control (LOC) were reported by Golding et al. [52]. In their study, an external LOC for mothers and to some degree, for fathers, was significantly associated with adolescent obesity at each point the measurement were made. Furthermore, adolescent LOC showed significant correlation with obesity from age 13. These finding are in line with the results of the study performed by Radcliff et al. among Latina youths [24]. In this study, higher participant IHLC was associated with lower BMI.

Our observations that only external HLC is significantly associated with nutritional behaviors may guide potential interventions addressing the health behaviors of adolescents. The fact that PHLC is related both to recommended dietary patterns and the consumption of fast food may indicate that such interventions should involve those people in the closest social network of adolescents, possibly family members and peers. The reports from implemented health promotion programs show that focus on the whole family of a young person may be a success factor [53,54]. Furthermore, interventions mediated by adolescent peers may also be effective in influencing their behaviors [55,56,57].

Interestingly, our study suggests that male students at secondary schools show more beneficial behaviors in terms of the number of meals consumed daily, the regularity of meals and the largest meal consumed during the day than girls. This observation is opposite to findings from studies performed in other countries and in Poland. It may also signal an ongoing change in the lifestyle of female adolescents and their proclivity toward more unhealthy behaviors.

There was no difference in the frequency of the consumption of fruit and vegetables and fast food between the genders. Neither the level of education nor parent marital status were predictors of the analyzed indicator nutritional behaviors. Respondents from urban areas with the highest populations were more likely than the inhabitants of rural areas to show unfavorable nutrition patterns. The respondents from households with the lowest number of inhabitants were more likely to adhere to regular meals than those from households with a higher number of inhabitants. As for independent variables reflecting the economic status of the respondent family, no consistent pattern of relationships was observed, apart from the level of expenditures on mobile phones. The respondents declaring the highest level of spending showed less favorable nutritional behaviors. Finally, more spent on the Internet was significantly associated with lower regularity of meals, lower consumption of fruit and vegetables, and more frequent consumption of fast foods.

In the last few years, several systematic reviews synthesizing observations on the sociodemographic and economic determinants of the nutrition behaviors of adolescents or similar age groups, e.g., young adults, have been published. Mohammadi et al. performed a systematic review to analyze the determinants of diet and physical activity of Malaysian adolescents [58]. They found that the diet of males was of lower quality and that their intake of energy and macronutrients was higher than for females. However, the significance of the confirmed association was rather low and not consistent. In another systematic review, Noll et al. assessed the determinants of eating patterns and nutrient intake among adolescent athletes [59]. They observed that nutrient intake was associated with sport modality but not with the age or sex of participants of the included studies. The authors of a systematic review published in 2020 identified 40 original studies assessing the diet of adolescents and young adults [60]. The results of these studies revealed a significant relationship between favorable dietary patterns, higher dietary scores, greater consumption of fruit, vegetables and dairy products, lower consumption of sugar sweetened beverages and energy-dense foods, and higher parental socioeconomic status, particularly higher education. In the adjusted model, we have not seen a significant relationship between nutrition behaviors and education level of parents among Polish secondary school students.

According to the review of Saha et al., predictors of higher fast-food consumption among college students in South Asia included being younger, being of a higher socioeconomic class, being overweight or obese, studying humanities, possessing low nutrition knowledge and being addicted to the Internet [61]. Our analysis also showed that higher consumption of fast food is associated with more intense Internet use.

The results of recent studies performed among adolescents in Poland confirm only some of our study’s findings. Drywień et al. reported that among Polish metropolitan adolescents, favorable nutrition patterns occurred more frequently among girls, among those with siblings, and among respondents with insufficient rather than sufficient nutrition knowledge [62]. In this study group, similarly to adolescents from southern Poland, mother’s education and household size were not significant predictors of nutrition patterns. Myszkowska-Ryciak et al. analyzed the relationship between gender and age and nutritional behaviors in a large group of 13–19-year-old Polish adolescents [63]. They observed a lower frequency of regular breakfast consumption for older groups of adolescents and for girls. Older age was also associated with less regular consumption of fruit and vegetables, dairy products, sweetened beverages, and fast food. Sweets were consumed more frequently by girls. In our study, we have used the student grade in secondary school as a proxy for the respondent age and this variable was not consistently associated with adolescent nutrition behaviors. However, we have observed that boys had more favorable nutrition behaviors than girls.

Overall, our findings could be useful in the development and implementation of nutritional interventions addressed to youths in southern Poland. They suggest that certain subgroups may require particular attention, e.g., adolescents living in larger urban areas or those attending vocational schools. It also seems that more intense use of modern technologies, as demonstrated by the expense on mobile phones or the duration of weekly Internet use, is related to negative nutritional choices and meal regularity. A positive association between prolonged leisure-time Internet use and unfavorable nutritional behaviors including lower consumption of fruit and vegetables and higher consumption of instant noodles, fast food, chips, crackers, and sugar sweetened beverages, has been recently confirmed on large sample of Korean adolescents [64]. The authors of this study also reported a difference in potential impact between leisure- and study-time Internet use on dietary risk behaviors. Specifically, longer study-time has not been linked to lower fruit and vegetable intake. So, it seems that the potential influence of Internet use is not straightforward and depends on the profile of the activities performed in cyberspace.

It is hypothesized that the mechanisms responsible for the higher prevalence of risky dietary behaviors among adolescent using digital media for longer periods of time include the exposure to food marketing or to social media content showing peers in popular sites, e.g., those serving fast food [64]. Another recent study performed on the multinational European sample even showed that digital media use is positively associated with a shift of taste preferences toward sweet, fatty, salty, and bitter food in children and adolescents [65]. Finally, the association between the prevalence of Internet addiction and unhealthy dietary behaviors has been also confirmed in a national sample of adolescents from Malaysia [66]. All these findings tend to indicate that the use of Internet and mobile phones in adolescence has become a serious public health challenge requiring attention and an appropriate place in national and regional health programs addressed to this age group.

### Limitations

Our analysis only included only selected indicator nutrition behaviors. We have included three variables characterizing dietary patterns of dieting and two variables reflecting the consumption of the specific types of food. Due to the broad scope of the survey, we have not been able to include more detailed assessment of nutrition behaviors in the analysis.

The study was performed in only one voivodship in southern Poland. So, extrapolating the extrapolation of obtained results to the whole population of secondary school pupils in Poland is not possible. On the other hand, the relatively large sample size of equal 2223 respondents assures that we have been able to ascertain the nutrition behaviors of youths in the above-mentioned part of the country.

Finally, we have applied a standard, 47-item version of the HLS-EU Questionnaire. We have assumed it to be an adequate tool for the subjects (secondary school representing the students at secondary schools in Poland, using 16–19 years old). However, some authors have developed and used the instruments for the assessment of HL that have been adjusted to the characteristics of the target population.

As for the regression analysis, we had to make some arbitrary decisions about dichotomizing the dependent variables used in the developed models. This could result in some loss of the information obtained from the survey. On the other hand, we could obtain a clear view of the effects of the set of the predictors used in the logistic regression modelling.

## 5. Conclusions

This study performed among secondary school students from a voivodship in southern Poland revealed rather complex patterns of relationships between predictor variables and the behaviors selected as indicators of nutrition patterns. The level of HL was associated with the types of consumed food but not with dietary habits. There was no significant association between IHLC and nutrition behaviors in the study group. More beneficial dietary patterns were seen among respondents with higher PHLC scores and less beneficial among those with higher CHLC. In our study, males showed more favorable dietary patterns than girls, opposite to the findings of other studies performed among Polish youths. Adjusted analyses have not shown parental level of education to be a significant predictor of student nutrition behaviors. The effect of other sociodemographic variables could be observed only in relation to selected independent variables reflecting nutrition behaviors. Among the variables reflecting respondent economic status, the level of expenditure on mobile phones was associated with unfavorable behaviors, as was the weekly duration of the Internet use.

Our results may serve as a recommendation for the development of health promotion interventions addressed to adolescents in Poland. They signal that some important changes in the perception of the impact of sociodemographic factors on the nutritional behaviors of this group should be taken into consideration, e.g., the presence of less favorable dietary patterns in girls. Furthermore, the role of HL should be reassessed and educational actions at schools or mediated by youth families must be extended to fill potential gaps, such as meal regularity or daily number of meals.

## Figures and Tables

**Table 1:** Characteristics of the study group.

Variable	Categories	%	n
Gender	girls	66.29	1457
	boys	33.71	741
Year in secondary school	1st	36.99	809
	2nd	28.81	630
	3rd or 4th	34.20	748
Type of school	general education	82.31	1829
	vocational training	17.69	393
Mother’s education level	lower than secondary	24.45	540
	secondary	37.35	825
	university	38.21	844
Father’s education level	lower than secondary	40.12	540
	secondary	33.14	825
	university	26.75	844
Marital status of parents	married	86.55	1918
	separated or divorced	10.24	227
	one or both parents deceased	3.20	71
Number of household members	<4	20.96	463
	4	33.26	734
	5	22.07	487
	>5	23.70	523
Place of residence	rural	51.53	1142
	urban ≤10,000	5.96	132
	urban >10,000 to 200,000	19.27	427
	urban >200,000	23.24	515
The size of the home (m^2)^	<50	9.68	213
	50–<70	15.41	339
	70–<90	11.59	255
	≥90	63.32	1393
Monthly spending on mobile phone (PLN)	<5	5.10	112
	5–<10	6.56	144
	10–<30	32.63	716
	30–<50	34.05	747
	≥50	21.65	475
Receiving external support (financial, material)	no	45.38	972
	yes	54.62	1170
Self-assessed economic situation	very bad, bad, or average	13.84	305
	good	53.40	1177
	very good	32.76	722
Number of books at home	≤25	12.37	272
	26–50	17.88	393
	51–100	27.53	605
	101–500	32.71	719
	>500	9.51	209
Weekly duration of Internet use (hours)	≤2	9.12	202
	>2–7	20.49	454
	>7–14	18.28	405
	>14–21	16.29	361
	>21–35	15.97	354
	>35	19.86	440
Daily number of meals	less than 4	29.84	658
	at least 4	70.16	1547
Regularity of meals	no	69.90	1500
	yes	30.10	646
The largest meal	dinner	84.84	1858
	other	15.16	332
The consumption of fruit and vegetables	less often than once daily	42.19	935
	at least once daily	57.81	1281
The consumption of fast food	at least a few times monthly	85.95	1903
	less often	14.05	311

Abbreviations: PLN—Polish zloty.

**Table 2:** Multivariate logistic regression models for the number of meals consumed daily and the regularity of meals.

Variable	Categories	Number of Meals Consumed Daily		The Regularity of Meals	
		OR (95% CI)	*p*	aOR	a95% CI
HL		1.01 (0.99–1.02)	0.303	1.00 (0.98–1.02)	0.989
IHLC		1.04 (0.92–1.17)	0.557	1.14 (0.99–1.32)	0.069
PHLC		0.89 (0.81–0.99)	0.028	1.16 (1.01–1.33)	0.039
CHLC		0.83 (0.75–0.92)	0.001	0.74 (0.65–0.84)	<0.001
Gender	female *				
	male	1.2 (0.99–1.46)	0.07	1.33 (1.05–1.67)	0.016
Year in secondary school	1st *				
	2nd	1.2 (0.96–1.51)	0.113	0.85 (0.65–1.1)	0.219
	3rd or 4th	1.19 (0.96–1.48)	0.121	0.84 (0.65–1.07)	0.161
Type of school	GE *				
	with VT	0.77 (0.61–0.97)	0.024	0.65 (0.47–0.89)	0.008
Education level of mother	primary or vocational *				
	secondary	1.27 (1–1.61)	0.047	1.18 (0.88–1.59)	0.277
	university	1.04 (0.83–1.32)	0.725	0.92 (0.66–1.29)	0.644
Education level of father	primary or vocational *				
	secondary	1.11 (0.9–1.38)	0.331	1.23 (0.93–1.61)	0.142
	university	0.96 (0.76–1.2)	0.692	1.3 (0.94–1.79)	0.110
Marital status of parents	Married *				
	divorced or separated	0.73 (0.55–0.98)	0.035	0.8 (0.54–1.18)	0.264
	one or both parents	0.89 (0.53–1.49)	0.664	0.56 (0.28–1.13)	0.107
Number of household members	<4 *				
	4	0.96 (0.75–1.24)	0.752	0.68 (0.5–0.92)	0.013
	5	1.13 (0.85–1.5)	0.396	0.67 (0.47–0.94)	0.022
	>5	1.11 (0.84–1.46)	0.466	0.77 (0.54–1.1)	0.152
Place of residence	rural *				
	urban ≤10,000	0.98 (0.65–1.48)	0.939	1.55 (1.00–2.41)	0.051
	urban >10,000 to 200,000	0.82 (0.64–1.04)	0.106	1.02 (0.77–1.36)	0.866
	urban >200,000	0.62 (0.49–0.77)	<0.001	0.74 (0.55–0.99)	0.041
Monthly expenses on mobile phone	≤5 PLN *				
	>5–10 PLN	1.18 (0.69–2.03)	0.542	0.88 (0.48–1.62)	0.679
	>10–30 PLN	1.07 (0.7–1.64)	0.753	0.83 (0.51–1.36)	0.464
	>30–50 PLN	1.37 (0.89–2.1)	0.156	0.88 (0.54–1.44)	0.61
	>50 PLN	0.9 (0.58–1.4)	0.644	0.59 (0.35–0.99)	0.047
Receiving external support	no *				
	yes	0.96 (0.79–1.15)	0.631	1.09 (0.86–1.36)	0.482
Self-assessed economic situation	worse than good *				
	good	1.22 (0.93–1.59)	0.155	0.93 (0.67–1.30)	0.678
	very good	1.22 (0.91–1.62)	0.179	1.13 (0.79–1.61)	0.516
Weekly duration of Internet use	not more than 2 h *				
	>2–7 h	0.91 (0.64–1.31)	0.625	0.84 (0.56–1.26)	0.401
	>7–14	1.23 (0.85–1.79)	0.276	0.99 (0.66–1.49)	0.973
	>14–21	1.17 (0.8–1.71)	0.422	0.92 (0.61–1.40)	0.709
	>21–35	1.07 (0.73–1.56)	0.73	0.64 (0.42–0.98)	0.041
	>35 h	0.8 (0.56–1.15)	0.235	0.5 (0.33–0.77)	0.002

Abbreviations: *—referential category of variable, aOR (95% CI)—adjusted odds ratio (95% confidence interval), *p*—*p*-value for a univariate or multivariate logistic regression model, VT—vocational training, GE—general education, div.—divorced, voc.—vocational, IHLC—internal health locus of control, PHLC—“powerful others health locus of control”, CHLC—“chance health locus of control”, PLN—Polish zloty.

**Table 3:** Multivariate logistic regression model for the variable related to the most abundant meal.

Variable	Categories	aOR	a95% CI
HL		1.00 (0.98–1.03)	0.744
IHLC		0.95 (0.79–1.13)	0.562
PHLC		1.06 (0.89–1.26)	0.500
CHLC		1.08 (0.92–1.27)	0.349
Gender	female *		
	male	1.37 (1.02–1.85)	0.040
Year in secondary school	1st *		
	2nd	0.70 (0.50–0.99)	0.041
	3rd or 4th	0.71 (0.52–0.98)	0.037
Type of school	GE *		
	with VT	0.59 (0.42–0.84)	0.004
Education level of mother	primary or vocational *		
	secondary	0.84 (0.57–1.22)	0.352
	university	0.70 (0.46–1.06)	0.094
Education level of father	primary or vocational *		
	secondary	1.02 (0.73–1.44)	0.902
	university	0.85 (0.57–1.26)	0.416
Marital status of parents	married *		
	divorced or separated	1.04 (0.66–1.65)	0.860
	one or both parents	1.38 (0.6–3.18)	0.446
Number of household members	<4 *		
	4	1.68 (1.15–2.45)	0.007
	5	1.19 (0.79–1.80)	0.404
	>5	1.52 (0.99–2.35)	0.057
Place of residence	rural *		
	urban ≤10,000	1.33 (0.72–2.43)	0.359
	urban >10,000 to 200,000	1.1 (0.77–1.58)	0.611
	urban >200,000	1.31 (0.91–1.88)	0.145
Monthly expenses on mobile phone	≤5 PLN *		
	>5–10 PLN	0.58 (0.26–1.30)	0.185
	>10–30 PLN	0.86 (0.43–1.71)	0.671
	>30–50 PLN	0.83 (0.42–1.66)	0.605
	>50 PLN	0.72 (0.35–1.46)	0.358
Receiving external support	no *		
	yes	0.89 (0.67–1.19)	0.445
Self-assessed economic situation	worse than good *		
	good	1.49 (1.01–2.18)	0.042
	very good	1.48 (0.96–2.27)	0.073
Weekly duration of Internet use	not more than 2 h *		
	>2–7 h	0.9 (0.55–1.49)	0.686
	>7–14	1.05 (0.62–1.76)	0.854
	>14–21	1.33 (0.77–2.30)	0.301
	>21–35	1.33 (0.77–2.29)	0.308
	>35 h	1.14 (0.68–1.93)	0.613

Abbreviations: *—referential category of variable, aOR (95% CI)—adjusted odds ratio (95% confidence interval), *p*—*p*-value for a univariate or multivariate logistic regression model, VT—vocational training, GE—general education, div.—divorced, voc.—vocational, IHLC—internal health locus of control, PHLC—“powerful others health locus of control”, CHLC—“chance health locus of control”, PLN—Polish zloty.

**Table 4:** Multivariate logistic regression models for the consumption of fruit and vegetables and the consumption of fast food.

Variable	Categories	The Consumption of Fruit and Vegetable		The Consumption of Fast Food	
		aOR	a95% CI	aOR	*p*
HL		1.02 (1.01–1.04)	0.003	0.98 (0.95–0.999)	0.048
IHLC		1.08 (0.95–1.23)	0.25	0.99 (0.82–1.19)	0.884
PHLC		0.88 (0.78–1.00)	0.051	1.49 (1.24–1.79)	<0.001
CHLC		0.90 (0.8–1.01)	0.071	1.11 (0.94–1.32)	0.230
Gender	female *				
	male	0.82 (0.67–1.02)	0.073	1.31 (0.95–1.8)	0.096
Year in secondary school	1st *				
	2nd	1.05 (0.82–1.34)	0.715	0.89 (0.61–1.29)	0.538
	3rd or 4th	0.91 (0.72–1.14)	0.399	0.73 (0.52–1.01)	0.061
Type of school	GE *				
	with VT	0.68 (0.52–0.88)	0.004	1.5 (0.98–2.32)	0.065
Education level of mother	primary or vocational *				
	secondary	1.08 (0.83–1.41)	0.565	1.05 (0.71–1.54)	0.819
	university	1.17 (0.87–1.57)	0.296	1.35 (0.87–2.10)	0.186
Education level of father	primary or vocational *				
	secondary	1.2 (0.94–1.53)	0.143	1.05 (0.72–1.52)	0.796
	university	1.38 (1.03–1.86)	0.030	0.76 (0.50–1.16)	0.197
Marital status of parents	married *				
	divorced or separated	0.9 (0.64–1.27)	0.547	0.94 (0.57–1.55)	0.813
	one or both parents	0.67 (0.38–1.18)	0.162	0.74 (0.35–1.58)	0.444
Number of household members	<4 *				
	4	0.8 (0.60–1.06)	0.119	1.33 (0.89–2.01)	0.167
	5	0.76 (0.55–1.05)	0.091	0.87 (0.56–1.36)	0.547
	>5	0.82 (0.59–1.14)	0.238	1.02 (0.64–1.62)	0.942
Place of residence	rural *				
	urban ≤10,000	0.91 (0.60–1.40)	0.682	1.04 (0.56–1.94)	0.890
	urban >10,000 to 200,000	0.74 (0.57–0.96)	0.025	1.25 (0.83–1.89)	0.277
	urban >200,000	0.91 (0.70–1.18)	0.478	0.83 (0.58–1.19)	0.315
Monthly expenses on mobile phone	≤5 PLN *				
	>5–10 PLN	0.7 (0.39–1.25)	0.228	1.58 (0.73–3.4)	0.243
	>10–30 PLN	0.67 (0.42–1.08)	0.102	1.70 (0.93–3.10)	0.085
	>30–50 PLN	0.83 (0.52–1.34)	0.446	1.64 (0.90–2.98)	0.109
	>50 PLN	0.60 (0.36–0.98)	0.041	2.22 (1.16–4.25)	0.016
Receiving external support	no *				
	yes	1.07 (0.87–1.32)	0.524	0.72 (0.53–0.98)	0.035
Self-assessed economic situation	worse than good *				
	good	0.90 (0.67–1.21)	0.487	1.25 (0.83–1.88)	0.292
	very good	1.18 (0.85–1.63)	0.324	1.12 (0.71–1.77)	0.616
Weekly duration of Internet use	not more than 2 h *				
	>2–7 h	0.85 (0.58–1.25)	0.417	2.09 (1.31–3.33)	0.002
	>7–14	0.93 (0.63–1.38)	0.712	2.52 (1.54–4.12)	<0.001
	>14–21	0.88 (0.59–1.31)	0.522	2.29 (1.40–3.77)	0.001
	>21–35	0.84 (0.56–1.25)	0.381	2.99 (1.79–5.00)	<0.001
	>35 h	0.66 (0.45–0.97)	0.035	4.21 (2.45–7.23)	<0.001

Abbreviations: *—referential category of variable, aOR (95% CI)—adjusted odds ratio (95% confidence interval), *p*—*p*-value for a univariate or multivariate logistic regression model, VT—vocational training, GE—general education, div.—divorced, voc.—vocational, IHLC—internal health locus of control, PHLC—“powerful others health locus of control”, CHLC—“chance health locus of control”, PLN—Polish zloty.

## Data Availability

The data that support the findings of this study are available on request from the corresponding author. The data are not publicly available due to privacy and ethical restrictions. The authors did not inform participants that public access to the survey data may be considered. Access to the data will be granted on a case-by-case basis, following a justified request after receiving consent from the Bioethical Committee at Jagiellonian University.

## Supplementary Materials

The following are available online at https://www.mdpi.com/article/10.3390/nu13124323/s1, Table S1: Univariate logistic regression models for the number of meals consumed daily; Table S2: Univariate logistic regression models for regularity of meals consumed daily; Table S3: Univariate logistic regression models for the variable related to the most abundant meal; Table S4: Univariate logistic regression models for the consumption of fruit and vegetables; Table S5: Univariate logistic regression models for the consumption of fast food.
