# Supplementary material for: Nutritional Behaviors, Health Literacy, and Health Locus of Control of Secondary Schoolers in Southern Poland: A Cross-Sectional Study
Source: Nutrients. 2021 Nov 29;13(12):4323. doi: 10.3390/nu13124323 (PMC8709351; doi:10.3390/nu13124323)
Supplement: Supplementary file 1 [file nutrients-13-04323-s001.zip › nutrients-1472969-supplementary.pdf]

Table S1. Univariate logistic regression models for the number of meals consumed daily.

Variable	Categories	OR (95%CI)	p
HL		1.01 (0.99 - 1.02)	0.303
IHLC		1.04 (0.92 - 1.17)	0.557
PHLC		0.89 (0.81 - 0.99)	0.028
CHLC		0.83 (0.75 - 0.92)	0.001
Gender	female*		
	male	1.2 (0.99 - 1.46)	0.07
Year in secondary school	1st *		
	2nd	1.2 (0.96 - 1.51)	0.113
	3rd or 4th	1.19 (0.96 - 1.48)	0.121
Type of school	GE*		
	with VT	0.77 (0.61 - 0.97)	0.024
Education level of mother	primary or vocational*		
	secondary	1.27 (1 - 1.61)	0.047
	university	1.04 (0.83 - 1.32)	0.725
Education level of father	primary or vocational*		
	secondary	1.11 (0.9 - 1.38)	0.331
	university	0.96 (0.76 - 1.2)	0.692
Marital status of parents	married*		
	divorced or separated	0.73 (0.55 - 0.98)	0.035
	one or both parents	0.89 (0.53 - 1.49)	0.664
Number of household members	<4*		
	4	0.96 (0.75 - 1.24)	0.752
	5	1.13 (0.85 - 1.5)	0.396
	>5	1.11 (0.84 - 1.46)	0.466
Place of residence	rural*		
	urban ≤ 10,000	0.98 (0.65 - 1.48)	0.939
	urban >10,000 to 200,000	0.82 (0.64 - 1.04)	0.106
	urban >200,000	0.62 (0.49 – 0.77)	<0.001
Monthly expenses on mobile phone	≤5 PLN*		
	>5 - 10 PLN	1.18 (0.69 - 2.03)	0.542
	>10 - 30 PLN	1.07 (0.7 - 1.64)	0.753
	>30 - 50 PLN	1.37 (0.89 - 2.1)	0.156
	>50 PLN	0.9 (0.58 - 1.4)	0.644
Receiving external support	no*		
	yes	0.96 (0.79 - 1.15)	0.631
Self-assessed economic situation	worse than good*		
	good	1.22 (0.93 - 1.59)	0.155
	very good	1.22 (0.91 - 1.62)	0.179
Weekly duration of Internet use	not more than 2 hours*		
	>2 - 7 hours	0.91 (0.64 - 1.31)	0.625
	>7 - 14	1.23 (0.85 - 1.79)	0.276
	>14 - 21	1.17 (0.8 - 1.71)	0.422
	>21 - 35	1.07 (0.73 - 1.56)	0.73
	>35 hours	0.8 (0.56 - 1.15)	0.235

Abbreviations: * - referential category of variable, OR (95%CI) – odds ratio (95% confidence interval), p – p-value for a univariate logistic regression model, VT – vocational training, GE – general education, div. – divorced, voc. – vocational, IHLC – internal health locus of control, PHLC – 'powerful others health locus of control', CHLC – 'chance health locus of control', PLN – Polish zloty.

Table S2. Univariate logistic regression models for regularity of meals consumed daily.

Variable	Categories	OR95%CI	p
HL		1.01 (1.00 - 1.03)	0.175
IHLC		1.17 (1.04 - 1.33)	0.009
PHLC		0.89 (0.81 - 0.99)	0.028
CHLC		0.75 (0.67 - 0.83)	<0.001
Gender	female*		
	male	1.41 (1.16 - 1.71)	0.001
Year in secondary school	1st *		
	2nd	0.94 (0.75 - 1.18)	0.598
	3rd or 4th	0.83 (0.67 - 1.04)	0.103
Type of school	GE*		
	with VT	0.64 (0.49 - 0.83)	0.001
Education level of mother	primary or vocational*		
	secondary	1.34 (1.05 - 1.72)	0.019
	university	1.28 (1 - 1.64)	0.052
Education level of father	primary or vocational*		
	secondary	1.27 (1.02 - 1.58)	0.036
	university	1.35 (1.07 - 1.7)	0.012
Marital status of parents	married*		
	divorced or separated	0.78 (0.57 - 1.07)	0.126
	one or both parents	0.70 (0.40 - 1.24)	0.221
Number of household members	<4*		
	4	0.86 (0.67 - 1.12)	0.266
	5	0.88 (0.66 - 1.17)	0.381
	>5	1.00 (0.76 - 1.31)	0.994
Place of residence	rural*		
	urban ≤ 10,000	1.36 (0.93 – 2.00)	0.116
	urban >10,000 to 200,000	1.03 (0.80 - 1.31)	0.836
	urban >200,000	0.76 (0.60 – 0.96)	0.023
Monthly expenses on mobile phone	≤5 PLN*		
	>5 - 10 PLN	1.18 (0.69 - 2.01)	0.538
	>10 - 30 PLN	0.96 (0.62 - 1.49)	0.865
	>30 - 50 PLN	0.95 (0.62 - 1.47)	0.823
	>50 PLN	0.58 (0.37 - 0.92)	0.020
Receiving external support	no*		
	yes	1 (0.83 - 1.21)	0.981
Self-assessed economic situation	worse than good*		
	good	1.05 (0.79 - 1.40)	0.742
	very good	1.33 (0.98 - 1.79)	0.063
Weekly duration of Internet use	not more than 2 hours*		
	>2 - 7 hours	0.75 (0.52 - 1.07)	0.109
	>7 - 14	0.92 (0.64 - 1.32)	0.651
	>14 - 21	0.91 (0.63 - 1.31)	0.606
	>21 - 35	0.62 (0.43 - 0.91)	0.014
	>35 hours	0.45 (0.31 - 0.66)	<0.001

Abbreviations: * - referential category of variable, OR (95%CI) – odds ratio (95% confidence interval), p – p-value for a univariate logistic regression model, VT – vocational training, GE – general education, div. – divorced, voc. – vocational, IHLC – internal health locus of control, PHLC – 'powerful others health locus of control', CHLC – 'chance health locus of control', PLN – Polish zloty.

Table S3. Univariate logistic regression models for the variable related to the most abundant meal.

Variable	Categories	OR 95%CI	p
HL		1.00 (0.98 - 1.02)	0.709
IHLC		1.06 (0.91 - 1.23)	0.466
PHLC		0.89 (0.81 - 0.99)	0.028
CHLC		1.02 (0.89 - 1.16)	0.776
Gender	female*		
	male	1.30 (1.00 - 1.68)	0.047
Year in secondary school	1st *		
	2nd	0.84 (0.62 - 1.13)	0.251
	3rd or 4th	0.74 (0.56 - 0.98)	0.037
Type of school	GE*		
	with VT	0.80 (0.60 - 1.08)	0.148
Education level of mother	primary or vocational*		
	secondary	0.93 (0.68 - 1.27)	0.632
	university	0.78 (0.58 - 1.07)	0.120
Education level of father	primary or vocational*		
	secondary	0.94 (0.71 - 1.25)	0.692
	university	0.81 (0.60 - 1.08)	0.147
Marital status of parents	married*		
	divorced or separated	0.79 (0.55 - 1.14)	0.210
	one or both parents	1.19 (0.58 - 2.41)	0.639
Number of household members	<4*		
	4	1.54 (1.12 - 2.12)	0.008
	5	1.13 (0.81 - 1.58)	0.475
	>5	1.54 (1.09 - 2.18)	0.015
Place of residence	rural*		
	urban ≤ 10,000	1.16 (0.69 - 1.96)	0.577
	urban >10,000 to 200,000	1.08 (0.79 - 1.48)	0.625
	urban >200,000	1.08 (0.80 – 1.45)	0.612
Monthly expenses on mobile phone	≤5 PLN*		
	>5 - 10 PLN	0.65 (0.32 - 1.35)	0.253
	>10 - 30 PLN	0.89 (0.48 - 1.65)	0.705
	>30 - 50 PLN	0.73 (0.40 - 1.35)	0.314
	>50 PLN	0.66 (0.35 - 1.23)	0.186
Receiving external support	no*		
	yes	0.80 (0.63 - 1.02)	0.078
Self-assessed economic situation	worse than good*		
	good	1.37 (0.98 - 1.91)	0.066
	very good	1.28 (0.90 - 1.83)	0.171
Weekly duration of Internet use	not more than 2 hours*		
	>2 - 7 hours	0.98 (0.63 - 1.54)	0.942
	>7 - 14	1.03 (0.65 - 1.63)	0.907
	>14 - 21	1.45 (0.89 - 2.37)	0.137
	>21 - 35	1.35 (0.83 - 2.20)	0.225
	>35 hours	1.03 (0.65 - 1.62)	0.905

Abbreviations: * - referential category of variable, OR (95%CI) – odds ratio (95% confidence interval), p – p-value for a univariate logistic regression model, VT – vocational training, GE – general education, div. – divorced, voc. – vocational, IHLC – internal health locus of control, PHLC – 'powerful others health locus of control', CHLC – 'chance health locus of control', PLN – Polish zloty.

Table S4. Univariate logistic regression models for the consumption of fruit and vegetables.

Variable	Categories	OR95%CI	p
HL		1.03 (1.01 - 1.04)	<0.001
IHLC		1.06 (0.95 - 1.18)	0.309
PHLC		0.89 (0.81 - 0.99)	0.028
CHLC		0.82 (0.75 - 0.91)	<0.001
Gender	female*		
	male	0.87 (0.73 - 1.04)	0.132
Year in secondary school	1st *		
	2nd	1.11 (0.9 - 1.38)	0.317
	3rd or 4th	0.91 (0.75 - 1.11)	0.365
Type of school	GE*		
	with VT	0.59 (0.47 - 0.73)	<0.001
Education level of mother	primary or vocational*		
	secondary	1.26 (1.02 - 1.57)	0.036
	university	1.67 (1.34 - 2.08)	<0.001
Education level of father	primary or vocational*		
	secondary	1.24 (1.01 - 1.51)	0.036
	university	1.59 (1.29 - 1.98)	<0.001
Marital status of parents	married*		
	divorced or separated	1.00 (0.76 - 1.32)	0.996
	one or both parents	0.68 (0.42 - 1.09)	0.111
Number of household members	<4*		
	4	0.84 (0.66 - 1.07)	0.155
	5	0.81 (0.63 - 1.05)	0.119
	>5	0.79 (0.61 - 1.02)	0.072
Place of residence	rural*		
	urban ≤ 10,000	1.24 (0.85 - 1.80)	0.264
	urban >10,000 to 200,000	0.86 (0.69 - 1.08)	0.189
	urban >200,000	0.997 (0.81 – 1.23)	0.976
Monthly expenses on mobile phone	≤5 PLN*		
	>5 - 10 PLN	0.94 (0.57 - 1.56)	0.822
	>10 - 30 PLN	0.98 (0.66 - 1.48)	0.941
	>30 - 50 PLN	1.17 (0.78 - 1.76)	0.442
	>50 PLN	0.80 (0.53 - 1.22)	0.302
Receiving external support	no*		
	yes	1.17 (0.98 - 1.39)	0.08
Self-assessed economic situation	worse than good*		
	good	1.08 (0.84 - 1.39)	0.559
	very good	1.58 (1.21 - 2.08)	0.001
Weekly duration of Internet use	not more than 2 hours*		
	>2 - 7 hours	0.86 (0.61 - 1.21)	0.382
	>7 - 14	0.87 (0.61 - 1.23)	0.424
	>14 - 21	0.84 (0.59 - 1.19)	0.323
	>21 - 35	0.88 (0.62 - 1.26)	0.497
	>35 hours	0.60 (0.43 - 0.84)	0.003

Abbreviations: * - referential category of variable, OR (95%CI) – odds ratio (95% confidence interval), p – p-value for a univariate logistic regression model, VT – vocational training, GE – general education, div. – divorced, voc. – vocational, IHLC – internal health locus of control, PHLC – 'powerful others health locus of control', CHLC – 'chance health locus of control', PLN – Polish zloty.

Table S5. Univariate logistic regression models for the consumption of fast food.

Variable	Categories	OR95%CI	p
HL		0.98 (0.96 – 1.00)	0.095
IHLC		0.99 (0.85 - 1.16)	0.919
PHLC		1.56 (1.35 - 1.81)	<0.001
CHLC		1.36 (1.19 - 1.57)	<0.001
Gender	female*		
	male	1.25 (0.96 - 1.63)	0.091
Year in secondary school	1st *		
	2nd	0.80 (0.59 - 1.09)	0.151
	3rd or 4th	0.69 (0.52 - 0.92)	0.012
Type of school	GE*		
	with VT	1.35 (0.96 - 1.9)	0.08
Education level of mother	primary or vocational*		
	secondary	0.94 (0.68 - 1.28)	0.676
	university	1.02 (0.75 - 1.40)	0.887
Education level of father	primary or vocational*		
	secondary	1.11 (0.83 - 1.49)	0.472
	university	0.84 (0.63 - 1.13)	0.251
Marital status of parents	married*		
	divorced or separated	0.93 (0.63 - 1.38)	0.725
	one or both parents	0.64 (0.35 - 1.17)	0.146
Number of household members	<4*		
	4	1.23 (0.88 - 1.72)	0.222
	5	0.94 (0.66 - 1.33)	0.714
	>5	1.17 (0.82 - 1.68)	0.385
Place of residence	rural*		
	urban ≤ 10,000	0.94 (0.56 - 1.58)	0.821
	urban >10,000 to 200,000	1.17 (0.84 - 1.64)	0.356
	urban >200,000	0.82 (0.61 – 1.09)	0.162
Monthly expenses on mobile phone	≤5 PLN*		
	>5 - 10 PLN	1.30 (0.69 - 2.45)	0.424
	>10 - 30 PLN	1.54 (0.93 - 2.54)	0.095
	>30 - 50 PLN	1.64 (0.99 - 2.72)	0.054
	>50 PLN	2.21 (1.28 - 3.81)	0.004
Receiving external support	no*		
	yes	0.74 (0.58 - 0.96)	0.021
Self-assessed economic situation	worse than good*		
	good	1.31 (0.93 - 1.86)	0.125
	very good	1.11 (0.77 - 1.60)	0.566
Weekly duration of Internet use	not more than 2 hours*		
	>2 - 7 hours	1.95 (1.30 - 2.92)	0.001
	>7 - 14	2.46 (1.60 - 3.77)	<0.001
	>14 - 21	2.11 (1.37 - 3.24)	0.001
	>21 - 35	2.31 (1.49 - 3.58)	<0.001
	>35 hours	3.98 (2.51 - 6.33)	<0.001

Abbreviations: * - referential category of variable, OR (95%CI) – odds ratio (95% confidence interval), p – p-value for a univariate logistic regression model, VT – vocational training, GE – general education, div. – divorced, voc. – vocational, IHLC – internal health locus of control, PHLC – 'powerful others health locus of control', CHLC – 'chance health locus of control', PLN – Polish zloty.
